# Out of the Darkness and Into the Light: Confronting the Global Challenges in Wound Education

**DOI:** 10.1111/iwj.70178

**Published:** 2025-01-12

**Authors:** Lisa Gould, Ira Herman

**Affiliations:** ^1^ South Shore Health South Weymouth Massachusetts USA; ^2^ Tufts University School of Medicine Boston Massachusetts USA

The global landscape of wound care is shrouded in shadows and plagued by inadequate education, inconsistent standards and a marked lack of understanding and confusion about how to treat wounds that impacts patients and providers. Despite the significant burden of chronic wounds on healthcare systems worldwide, the field remains under‐resourced, overlooked and poorly represented within the curricula of modern‐day professional school training. It is time that we raise awareness locally and globally to shed light on the pressing issues that hinder progress in wound care and advocate for a more enlightened approach that prioritises education, innovation and patient‐centric care.

While non‐healing, hard‐to‐heal or ‘chronic’ wounds represent a ‘silent pandemic’ that is seemingly in lock step with the ever‐growing increase in diabetes and obesity, it is well documented that major gaps in basic wound education and management exist for both primary care and front line clinicians [1, 2, 3, 4]. These gaps in knowledge and care are transdisciplinary and global, affecting the acute and post‐acute care settings. However, with advancements in non‐invasive diagnostics that enable personalised and actionable evidence‐based treatments, coupled with enhanced education and early intervention there is enormous potential to (i) prevent the development of chronic wounds, (ii) reduce hospitalizations and (iii) significantly reduce the financial and emotional toll of this pandemic on the globe's healthcare delivery systems [5]. Thus, it is imperative that trainees across the care continuum receive all the tools needed to optimise wound care so that the right treatment for the right reason and at the right time is delivered to each and every patient, regardless of wound type, aetiology or socio‐economic status. A comprehensive curriculum must include basic education about the science of wound repair and regeneration, along with a standardised approach to basic wound care, while emphasising the cost‐savings and benefits of deploying interprofessional teams to advance the care of these complex and costly cases. A vast literature confirms that trainees rank their knowledge about wound care as low, yet desire to have practical skills that will help them care for their patients. The medical curriculum is at a crossroads, as the amount of information exceeds capacity in terms of time and expertise. This has restricted the ability to incorporate basic education about wound pathophysiology, wound diagnosis and wound treatment. Despite the gaps and inconsistencies, there are stories of successful expansion of the knowledge base and integration of wound care into the healthcare professionals' basic curriculum. This perspective article examines the challenges and solutions to this pressing problem.

At the May 2024 Annual Conference of the Wound Healing Society (WHS), the International session explored global solutions to wound healing education. Susan Volk, VMD, PhD, DACVS, chair of the WHS International Relations committee, Ira Herman, PhD and Lisa Gould, MD, PhD moderated the session that included presentations from recognised experts in wound education and interviews with clinicians around the globe who discussed their experience, challenges and novel solutions to providing basic wound education to clinicians and patients.

The session opened with a brief animated video, ‘Wound Healing Lessons from the Home: Intergenerational Learning Saves Limbs and Lives’. The video, crafted by Ira Herman and Lisa Gould depicts an 11‐year‐old girl who advocates for her grandmother who is suffering with the complications of diabetes and a long‐standing foot ulcer.

The animation demonstrates the power and potential of intergenerational learning for patients being cared for at home and sends the message that early diagnosis and treatment can save limbs and lives, while illustrating the critical need to inspire the next generation of clinicians and researchers. The video closes with a call to action, presenting the staggering facts about the current ‘pandemic’ facing all those in need of healing. In the end, it is all about education.

This was followed by a combination of live presentations and video interviews that highlight the problems encountered across the globe along with novel solutions to expand wound education for all learners.

## 1 Project ECHO, R. Gary Sibbald, BSc, MD FRCPC (Med, Derm), MACP, FAAD, MEd, FAPWCA

Dr. Gary Sibbald, a dermatologist and internist, internationally known for his expertise in wound care and education, is the project lead on ECHO (Extension for Community Healthcare Outcomes) Ontario Skin & Wound that virtually reaches a wide variety of healthcare professionals including Northern and Indigenous centres. Project ECHO emphasises interprofessional collaboration, early screening and patient education. Using a spoke and hub model, there is an emphasis on educating healthcare professionals in practice, developing interprofessional teams, use of multimodal didactic methods, case‐based interactive learning and virtual skills training, leading to evidence‐based care management plans with outcome evaluation. The project has trained more than 450 healthcare professionals in 96 health care organisations and provided team consultations to more than 120 complex patients. More than 90% of participants said the learning met their needs and 87% changed their practice. The project emphasises treating patients in their communities, by ‘moving knowledge, not patients’ [6].

## 2 The Importance of Wound Diagnosis for the General Practitioner, Kirsi Isoherranen, MD, PhD

Dr. Kirsi Isoherranen, president of EWMA (European Wound Management Association) and chief physician of the Helsinki Wound Healing Centre in Finland described two cohort studies, one in Finland and one in the United States, that highlight the diagnostic delay between primary care physicians and wound care team physicians. In these studies, as many as 26% of patients seen by general practitioners lacked a diagnosis, while less than 2% of wounds seen by the wound team lacked a diagnosis [7, 8]. This leads to delays in treatment, particularly as patients in clinical practice are becoming more complicated. She recommended the use of checklists and described a validated digital checklist called the Wound Navigator that provides structure to support clinical decision making that is particularly helpful when primary care physicians are faced with complex wounds [9]. In addition, she outlined the use of mnemonic aids to facilitate diagnosing wounds [10, 11]. The importance of early diagnostics in lower leg ulcers has also been highlighted in a recent EWMA document [12].

## 3 Voices From Around the Globe

To amplify the global impact of wounds and wound education, five clinicians described successful programmes and the challenges that they have encountered in their respective countries.

Anthony Sassi, PA‐C works in a Federally Qualified Health Center in rural Vermont, US. He described the lack of basic wound education for physician assistants in training that led him to seek an elective outside of the curriculum. He is a proponent of early training and exposure prior to employment for all clinicians, but also noted the unmet need for clinicians practicing in rural settings where resources and specialists are limited.

Cornelia Erfurt‐Burge, MD is a dermatologist at the University Hospital in Erlangen, Germany. She explained that she was not exposed to chronic wounds in her basic medical education but is now employed in a clinic where she treats patients with complex wounds. That prompted her to survey the medical students, who noted that they had no lectures about wound care. She developed a digital education module about wound care that is offered as an elective in dermatology. She is now working with general practitioners to integrate an obligatory lecture about diseases of the leg into their curriculum. Her university also offers an interprofessional skills course in which medical students and nurses work side by side, learn from each other and learn the relevance of wound care regardless of future specialty [13].

Terry Swanson, NP was one of the first nurse practitioners in Victoria, Australia. She has made wound care and wound education a focus of her profession and provides consultation services across Australia. She explained that while general practitioners are the medical gatekeepers in Australia, they do not have the knowledge or skills to treat patients with wounds. She discussed the work that Wounds Australia did to bring the Australian Medical Association (AMA) to the realisation that general practitioners require this education. The AMA is now funding an initiative to provide education for general practitioners that will allow them to recognise and provide the specialised care that patients with diabetic foot ulcers require [14].

Roch Christian Johnson, MD, MSc, PhD is an epidemiologist and infectious disease specialist who discussed a novel project in Benin, West Africa to provide wound education for patients with Buruli ulcers and leprosy. He highlighted the integration of the education team, including a social anthropologist, to explore current wound practices and provide basic wound education for the village caregivers. In depth discussions about ‘do's’ and ‘don'ts’ led them to identify basic principles of wound care that would fit with the practices and social structure of the villages. From this, they developed practical tools and methods to provide wound education and community awareness. He discussed the success with basic wound hygiene and the challenges of sustainability [15, 16].

Simone McConnie, BScPod Med, MChS HPC is the Caribbean Regional Coordinator of Dfoot International. Dfoot was developed as an outgrowth of the successful Step by Step project in Tanzania to reduce amputations with screening and prevention programmes for patients with diabetes and diabetic foot ulcers. She emphasised the great need to get buy‐in from the government, highlighting the success of the programme in Domenica and the need for sustainability that requires ongoing training and resources for the continuum of care [17, 18].

Lessons learned from these interviews include (1) the gravity of having wounds, especially foot wounds, in undeveloped countries, (2) the need to integrate into the local culture, (3) keeping wound care basic but evidence‐based, (4) involving local leaders, whether that be in the University, the Minister of Health or a major medical association and (5) the benefit of government funding for sustainability. (The entire video may be viewed at the following link: https://www.youtube.com/watch?v=s0t1‐nfHSwU. A transcript is included as a Supporting Information).

## 4 Discussion

**FIGURE 1:**
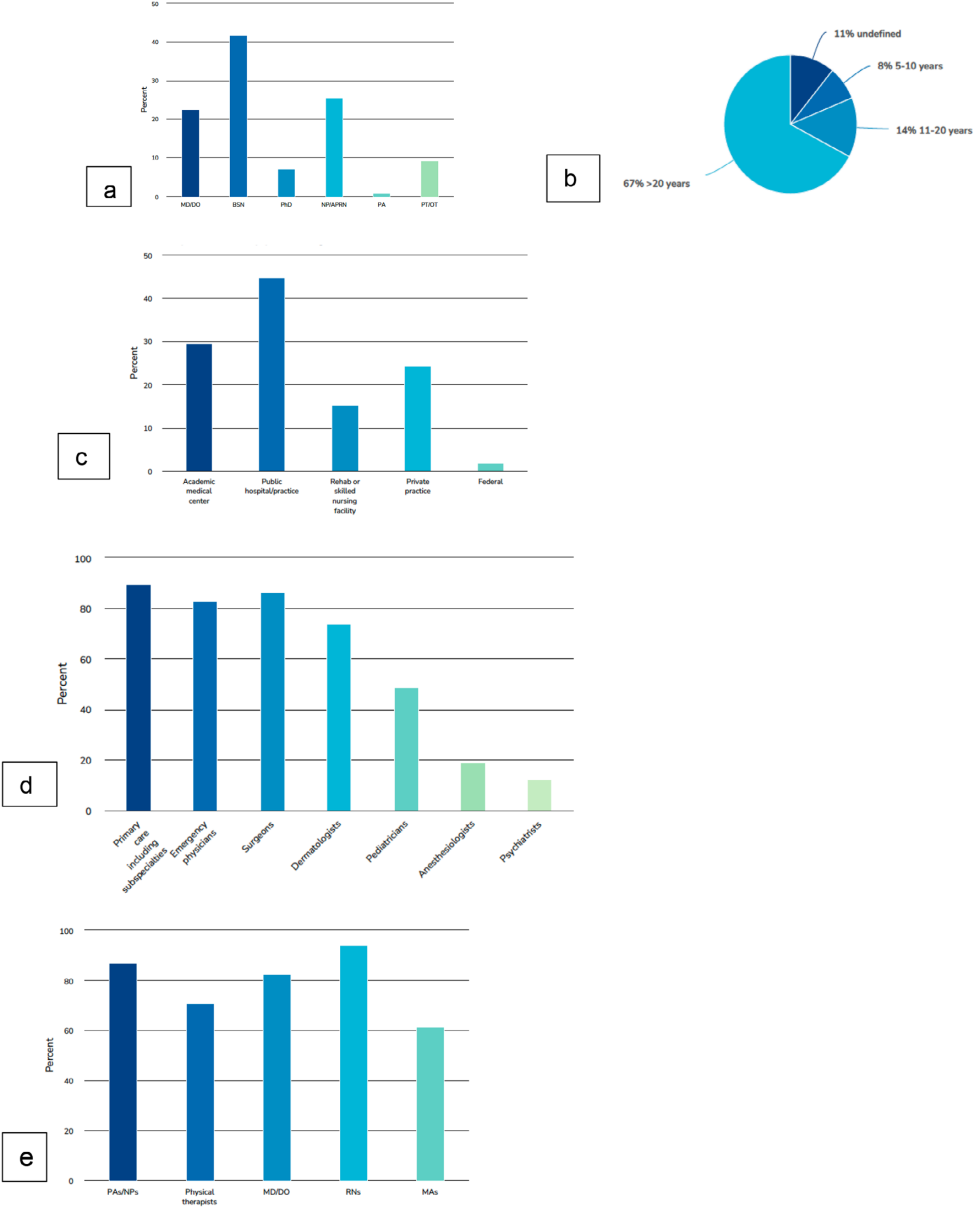
Pre‐conference survey with 102 respondents of whom 67% have been in practice for more than 20 years. (a) Credentials of the respondents. (b) Years in practice of respondents. (c) Respondents' practice location. (d) What specialties need to have basic wound education? (e) Which professionals or trainees should receive basic wound education?

An online survey conducted prior to the 2024 Wound Healing Society meeting confirmed that basic wound education should be transdisciplinary and interprofessional (Figure 1). However, wounds have no home in the usual systems‐based curriculum and cross so many disciplines that there is no ‘ownership’. It is possible to turn this into a positive attribute. For example, in the basic science curriculum when students learn the basics of inflammation it would be logical to make the connection to inflammatory ulcers, including the skin and gastrointestinal tract, the microbiome and the pharmacology of anti‐inflammatory drugs including monoclonal antibodies. The pathophysiology of diabetic wounds also addresses inflammation and is a perfect platform for reviewing vascular and neuroanatomy, pathophysiology of advanced glycation end products, renal physiology, endocrinology, nutrition, microbiology, foot anatomy and biomechanics of ambulation. This same disease state can lead to discussions of social determinants of health, health economics, population health and understanding health insurance programmes. All based on one wound.

## 5 Conclusions

The goal of the International WHS session was to bring together passionate educators, innovators and practitioners, all of whom are willing to push the envelope and demand basic wound education for all clinicians and caregivers, current and future. The session illustrated pilot projects that can be utilised to support a larger global strategy. Given the growth of this ‘silent epidemic’ and the under‐recognised disability of our patients, it is imperative that we work together to bring basic wound education to the forefront of medical education.

## Conflicts of Interest

The authors declare no conflicts of interest.

## Supporting information


**Data S1.** Video transcript (attachment).
