# Correction to “Out of the Darkness and Into the Light: Confronting the Global Challenges in Wound Education”

**DOI:** 10.1111/iwj.70195

**Published:** 2025-01-23

L. Gould and I. Herman, “Out of the Darkness and Into the Light: Confronting the Global Challenges in Wound Education,” *International Wound Journal* 22 (2025): e70178, https://doi.org/10.1111/iwj.70178.

The author information needs to be changed to add in secondary affiliation for Ira Herman and to make him co‐corresponding author.

Tufts University School of Medicine, Boston, Massachusetts, USA and Chief Science Officer, Tissue Health Plus, USA.

Email: ira.herman@tufts.edu

We apologize for this error.

