# Supplementary material for: Out of the Darkness and Into the Light: Confronting the Global Challenges in Wound Education
Source: Int Wound J. 2025 Jan 12;22(1):e70178. doi: 10.1111/iwj.70178 (PMC11725353; doi:10.1111/iwj.70178)
Supplement: Supplementary file 1 — Data S1. Video transcript (attachment). [file IWJ-22-e70178-s001.docx]

Global Education Video Interview Transcripts (edited for clarity)

**Anthony Sassi,PA-C rotated for 5 weeks with Dr. Gould as an elective clinical rotation in the last year of his PA training. He now works at a Federally Qualified Health Center in rural Vermont. In this video he discusses the need for wound care education as a trainee and also as a practicing clinician in a rural community.**

L Gould: Anthony, can you tell us a little bit about yourself and what motivated you to seek

wound care training during school?

A Sassi: Sure. Well, I'm a ninth generation Vermonter. And I always wanted to work primarily in primary care and family medicine, in a more rural setting. So, having seen a bit of that firsthand, having lived and grown up in a rural area, you know, I understand how there's such a need for wound care. And I thought it would be good to kind of advance those skills because I didn't feel that I necessarily had the best grasp of those.

And so that's why I had requested to come work with you. And that was a great experience.

L Gould: Did you get training or any wound care experience in PA school?

A Sassi: I did receive some, but I would say it was relatively minimal. From what I

remember, the bulk of our wound care came in one longer lecture. It may have been broken into two parts. I can't quite remember, but I believe the bulk of it was given by a P.T. And it was at the end of our training, and I really wish it had been earlier so we could have incorporated it in the clinicals a bit more. We did have some.

L Gould: Was that didactic or did you have hands on?

A Sassi: So it was it was all pretty much didactic.

L Gould:Okay. Now that you're in the workforce, I'm sure you're seeing some wounds and have seen other clinicians treating wounds. From your perspective, what could be done to improve wound care education for people like you or for other people out in the field?

A Sassi: Sure. You know, thinking back on my own training, I feel like it would have been very beneficial to have some exposure to wound care early in the curriculum. As a PA, for the first year or so, is all pretty much didactic, and we didn't really cover a whole lot of wound care in that time. I think that if it had been spread out more through the curriculum of my entire training, it would have actually been more helpful because I could have applied it at those stages of knowledge. But as it was, it was more kind of all at the very end of the training.

L Gould: And now that you're in the workforce, would it be valuable to have additional training, to help with what you're seeing?

A Sassi: Yes. Most definitely. I think that one can always continue training and learning. And I think that as a rural primary care provider right now, I'm seeing a lot of wounds that I

wish I had more exposure to prior to this job. You know, I think that my time spent in training with you, for example, offered a vast amount more than what I received in

my entire training. So I think it would be very beneficial. And there's such a need in the rural setting that I work in.

L Gould: What tools would you use, say, now that you're practicing?

Would online training be beneficial? Would, say, a regional Hands-On be beneficial? What would work best with your work?

A Sassi: I think online would be probably the most convenient for providers in the setting that I am in. I think that it would have more wide-reaching effect for a lot of us. However, I definitely feel like the hands-on component shouldn't be left out. So I think a mix of those things would be probably the most beneficial. But I think it also depends on the stage you are at.

L Gould: Are you also working with MDs who are doing some wound care?

A Sassi: I do work with some MDs who do wound care. I work with a nurse practitioner who is wound care certified. So we we're probably one of the largest FDQHS in Vermont where I work. And so we actually see quite a lot of wounds that come through. The one thing that I do notice a fair bit is that even some of the docs who do this, they do tend to send a lot of

wounds to more major hospitals, which are quite a ways away, just because they're not as comfortable with the wound care aspect of it.

L Gould: You preempted my question. I was going to ask where they send them when it's something that they can't manage.

A Sassi: So larger hospitals and then that's not so convenient for the patient if they have to travel, right? Yeah. So typically for us, we're in central Vermont, so they end up going to the University of Vermont Medical Center or Dartmouth, typically speaking. And, you know, for most of our patients, that's an hour and a half or two hour drive. And I've even run

across patients who have deferred wound care because they don't have transportation, for example. So it just highlights how important it is for wound care to be integrated into care, I think in rural areas.

L Gould: That's a good point. If they get care at the university hospital, would they then come back to your clinic for follow up.

A Sassi: They do sometimes especially if transportation is an issue. Or depending where they're at in their wound care. Sometimes that care is retained at those hospitals. Other times it is sent back to us for a variety of reasons, like I said. So it's a little dependent. I feel that there's a lot we probably could manage that gets sent to these larger facilities. And if we had more training where we're a bit more comfortable overall as a group of providers.

L Gould: Do you have any, podiatrists in your group that can help with the foot wounds?

A Sassi: Not in our group specifically. But there are several podiatrists at our local community hospital about 10 or 15 minutes away. And they're quite helpful.

L Gould: What kind of wounds have you been seeing. What are the conditions that you've been treating?

A Sassi: All kinds. A lot of pressure ulcers, a lot of illnesses and ulcers. Wounds from infections. There's a lot of farmers and more blue collar workers in this area. So they're not always the most inclined to come to the doctors and miss work. So sometimes when they come in their wounds are a little bit more advanced. From an injury or whatnot. So kind of a mix of everything I'd say.

L Gould: Do you have any other advice about education? How might we get it into the curriculum? Any ideas there?

A Sassi: I think approaching it as kind of an ongoing, integrated practice in a curriculum is probably the biggest thing that I think would be helpful. You know, for me, speaking personally, I think that having it in stages. So when I first started my training, for

example, obviously I didn't know all of the ins and outs of a lot of these, you know, more pathological aspects. But as I went, it would have been nice to kind of integrate wound care at the level I was learning. And I think that would help me really to solidify more of this information.

L Gould: So you got basic pathophysiology early on. And then you would want to be able to build on that as you see more patients. And then now as a practicing clinician there would be some additional materials or even some things to say brush up on that you might not have paid attention to because you didn't know the importance of.

A Sassi: Yeah, yeah. No for sure. And I think, you know, now that I am being the provider in this setting and I'm very hands on, some things that I never really thought as much about, even just basic dressings: Knowing differences, knowing what's available and cost and all those things play a huge role. So there's a lot to it. And I think that there's a lot that could be employed in a curriculum for PAs or other providers.

L Gould: That's really helpful. Thank you for providing that information.

**Transcript of Video interview (edited for clarity) with Cornelia Erfurt-Berge, MD, dermatologist at the University Hospital in Erlangen, Germany. She describes her interest in wound care and wound education, including implementation of wound education for medical students and primary care clinicians at her university. She highlights how to make wound care relevant to the student or general practitioner and emphasizes the value of a team approach in wound care but also wound education.**

L Gould: Cornelia, can tell me a little bit about yourself and how you got involved in wound care?

C. Erfurt-Berge: Yeah. So my name is Cornelia Erfurt-Berge, and I'm a senior

dermatologist at the university hospital in Erlangen, it's in the south of Germany.

I'm actually working for 20 years now, in dermatology. And I started to work with patients with chronic wounds quite early in my career, by incident at that time, but my interest had grown and grown. And so, over time, we initiated a certified wound center, which I'm now the head of and I specialize in addition to dermatologic surgeries or I could dive deeper into skin grafts, debridements and things like that.

And I'm also the educational coordinator at our university clinic. So I plan all the regular curricular learning phases for the medical students, but also some specialties.

L Gould: That's great. That's very busy. Before you started, was there education for

medical students in wound care?

C. Erfurt-Berge: No when I think I of my studies myself, I never heard about chronic wounds or anything like that.

So as I said, it was by incident that I started to work in this special outpatient department at that time, and I would never have thought of the connection between dermatology and chronic wounds, for example. Here at the clinic, we started with a survey among our students. When they are here, in the dermatological lecture, they are in the fifth year of six years in total, in Germany. So they are quite far in their studies. And we just asked them: Have you ever had any lectures about patients with chronic wounds or wound care in general? And the answer was overall, no. They had small incentives in surgery, for example, or diabetologia, but never structured.

So these results motivated us to go further into this topic. And I'm also an active member of the executive board of the ICW, the Scientific Society for Wound Healing in Germany. So I tried to bring this topic into this group of specialists. And I started asking my colleagues whether they offer a specific lecture or something about wound care. And this was mainly all the faculties, just as elective subjects, but not in the regular curriculum.

L Gould: So from that, have you been able to bring wound care education into the general curriculum, or is it still considered…

C. Erfurt-Berge:…this is still working on, here.

No. I started with some elective subjects. As you already have read in my article, we started with one, single case-based learning model, which we could offer, digitally.

This was before Covid times. So we started with this, and because I was the coordinator for educational projects, I brought in the topic of wound care in this digital project. And later on we developed, this first module. But so far I can only offer as an elective subject. But this semester I started to cooperate with other specialties like the general practitioners and we will offer a regular lecture. It's called ‘The diseases of the leg’. And there I will bring wound care also into this lecture. And this one is in the regular curriculum, and it's obligatory for all students in their third year.

L Gould: That's a good start. It is very difficult to get onto the curriculum. We noticed that the curriculae are full. So because of that, online training has become another avenue to get in. I noticed in your article that most of the opportunities were voluntary, but you had a pretty high number of students who took the course. How do we encourage our busy students to participate?

C Erfurt-Berge: Do you see, I have to smile about this question because we used a little trick there because we wanted to accomplish this project, scientifically.

So we needed a high number of data. And we lured them with a little reward, which was an extra point for the final exam, for the final written exam. And when they completed the online module, then they got this extra point. So it's the problem, that you are pointing to - that it is really hard to convince them to do anything extra or to get their interest.

And we often discussed about this problem in our team, because also in the elective subjects, they don't follow that so regularly or they complete all the modules and we have to motivate them on and on. I think we have to focus to let them see the relevance for their future life as a doctor independent of the specialty they will choose later on. And this is where I started with the general practitioners or in this lecture to show the students that chronic wound care can meet them in any specialty: Is it in private practice or as a

general practitioner, even, let's say, as an eye doctor in the clinic, you could have a patient with a leg ulcer, and you are a doctor and you have to say what is done next.

L Gould: That's true. I mean some of our patients don't see their general practitioner and they may only see a specialist for a couple of years.

C Erfurt-Berge: Yeah, and we are often discussing, at least in Germany, how to bring more attendance for wound care or these special patients to the general practitioners, or how we can make it that they send the patients earlier to our specialized wound centers.

And so this is an ongoing discussion about general practitioners who already are doctors. And my thought was always, why don't we start a step earlier with the students? Just to raise this awareness for the topic.

L Gould: No, I think that's very valuable.

Have you been able to follow up? I noticed that the skills was taught by video.

Have you been able to follow up on the students‘ actual skills and the implementation of their wound skills?

C Erfurt-Berge: Unfortunately, not in a scientific way. We discussed about this, but this would have been a project, so big that we couldn't do this in our very small team. And we learned that it's very dependent on manpower and financial support.

But we use, for example, these short videos about compression or bandaging or about measuring the ankle-brachial index for our students. Here in the clinic, when they start an internship, they are able to use these videos before they perform the measurement for the first time. And there we get really good feedback and we see a good outcome, but we haven't observed it in any scientifical or evaluated way so far.

We’re still thinking about this, but to perform it with this number of students would be a very large project. And yeah, it's manpower which is lacking here.

L Gould: Always. Outside of the course, are those videos available to other practitioners?

C Erfurt-Berge: Not so far. Of course. I can share them with whoever wants to use them. We are planning on such a project with our wound healing society to use them for official courses or something like that. But so far they're only shared on a personal way. But I'm open to this -just contact me.

L Gould: In Germany - so we know that in the UK, most of the wounds seem to be seen by the tissue viability nurses. And maybe don't get to a doctor until it is something

that really can't be handled by the nurse. What is the system like in Germany?

C. Erfurt-Berge: I think it's not completely comparable. But also here are specialized wound nurses who are working very close, for example, with the general practitioner. And their responsibility for the treatment is so far (this is just under change at the moment in

Germany) but so far responsibility is on the doctor who is maybe not that much specialized in wound care as the nurse, but he has the responsibility to say this dressing is correct or not. And this is a point where I talk to my medical students and tell them they have to know about wound care, because they will be responsible for the decisions. But as I said, this is under change at the moment. So more responsibility is brought to the nurses, which is quite good. So we may be shifting to a model like in the US.

L Gould: Yeah - it requires a team.

C Erfurt-Berge: Yeah, we also offer for example, practical skills course in our skills lab here at the University Hospital and these are interprofessional courses. They are always half and half in the group medical students and nursing students. And they're working together. And the medical students, they learn, okay, the nursing students may have more knowledge about wound care than I have.

L Gould: That's excellent. And I think that's a really good point. And, certainly true in areas in the United States and others that nurses get more training, but not across the board. So we need to make sure that everybody gets it. So we know that you're still in the area of trying to expand into the general practice education. Do you think that there is a future to be able to integrate into the medical school curriculum? Do you think it's well received by the general practitioner?

C Erfurt-Berge: Yes it is. You have to find cooperations with other people, doctors who are

interested in education. I'm currently working on this. Because we have at least one person in every clinic who is responsible for the education. So I try to reach out for them and to find common topics like wound care to integrate in the curriculum. For example, I

could imagine that not only for the general practitioners, but also in microbiology or palliative medicine or surgery, of course, could be my further destinations to infiltrate the education with wound care. But it's also always a personal thing. There has to be one person who wants to do it and who wants to invest time. We are doing all these projects outside of our regular working time, to be honest. But yeah, it's fun to work with

the students and to see that we bring something forward.

L Gould: This is really helpful. I think, that one of the things that we see is that there's a passionate person at one institution and so we get really good training in certain places but then it doesn't translate to others. So we're seeing that in the United States. I think our session is showing that there are some amazing islands of excellence. But we still need to reach a broader base. If we were to have, say, a global education project that provided high quality materials online and then perhaps regional areas that could augment that with skills, do you think that would be accepted?

C. Erfurt-Berge: Yeah, I think this would be highly accepted. And we could have an impact on, let's say, higher positions like, politics.

For example, in Germany, there are changes at the moment to change the medical curriculum in the next 2 to 5 years. So this is a chance to get in there with our topic. And this cannot be done by a single person or a single project. So these cooperatives would be perfect to have this political impact and to share our projects and our outcomes, our videos, whatever. We don't all have to reinvent the wheel at any single place. And we could exchange and this would be really highly appreciated.

L Gould:I think you're right. I think pretty much anyone who has done formal studies, their data demonstrate the same thing, so we can use each other's studies. And try to further the wound care environment.

Are there any other ideas that you have for bringing wound care to the masses? Because that's what we're really trying to do: to educate people that are not necessarily going to do it as their specialty, but they are going to face wounds.

C. Erfurt-Berge: I think we have to start with basic knowledge and to show them the relevance for their future work, maybe the personal relevance. We can say this might be your grandma with the leg ulcer. So we have to catch them somehow. And just tell them that they can be approached for wound care in any specialty and that they have to have a basic knowledge. In Germany, with the ICW we just started a project to develop learning goals in wound care; definite learning goals, which we can integrate in the regular curriculum.

And we all have to put our motivation and power together to work on this topic.

**Transcript of Video interview with Terry Swanson, Australia, edited for clarity**

**Terry Swanson, NP was endorsed in 2004 as one of the first Nurse Practitioners in her state and with a specialty of wound management in Australia.  Terry was admitted as a Fellow of the Australian Wound Management Association in 2010 for her significant contribution to wound management at a state, national and international level. She describes the Australian Medical Association’s recent initiative to implement a federally funded wound care strategy that includes wound education for general practitioners.**

L Gould: Terry, it's so nice to meet you. Will you please tell me a little bit about yourself and the impact of chronic wounds in Australia?

T Swanson: Thank you for the interest Lisa. So I immigrated to Australia in 1988. So the accent you can tell is from the United States, from Illinois. The burden of chronic wound care in Australia: We have approximately, according to the literature, about 450,000 patients per annum, that takes up about 2% of our healthcare budget per annum, which equates to about 3 billion. So with that burden, done by multiple bodies to explore that,

but the most exciting news has come from the Australian Medical Association with their initiatives. And so a couple years ago they put to the federal government their solutions to chronic wound burden in Australia. Because Australia doesn't have

a lot of reimbursement schemes. We do have the Medicare Benefits Scheme, which the doctors use their numbers to charge for their consultations, minor

procedures. But a couple of years ago, under a different government, they lost their wound care and their numbers. There's also pharmaceutical benefits scheme, but that doesn't provide subsidies for wound consumables. There's a work force initiative that helps GP's get practice nurses that are subsidized. And the Department of Veterans Affairs has a scheme, depending on their level of service, whether all or partial of all their consumables and care is provided for free. But the five point scheme that the federal government awarded to the AMA is still in evolution, still not fully funded and determined, but the scheme is really good. So one that they're currently working on is a funded wound consumable scheme to subsidize wound dressings. There's a committee looking at what would be subsidized, how the plan will be implemented, how it gets to the consumer, accountability, all that stuff to be determined. To implement a stepped model of care: So starting with the practice nurses, the GPs - right now, there's no MBS funding for them to do a holistic assessment, and not be out of pocket and then implement that subsidized care. And then after a given time, if the patient still doesn't progress, then they are referred off to specialists and then coordination of all that care.

LGould: I’m sorry to interrupt you - don't they have a plan for education and training the people?

T Swanson: Yeah. So that's number four. But this is a foundation of this and then a creation of new wound specific MBS. And then the fourth one is the National Education and Training for that. And so then the fifth part would be that all GP's who access the MBS and the consumable scheme would have to have required or mandatory education and training, and then the fifth would be the improved coordination of all that wound care. Right now we're all working in silos with the AMA, The Royal College of General Practitioners, the Australian Wound Management Association, or the Australian Wound and Skin Alliance. We're all working in silos. So trying to coordinate that sort of initiative

LGould: Sounds very similar to the United States. Right?

T Swanson: But what that mandatory education would be is yet to be determined. But I think it's great that we're looking at more national strategies. We don't have as many states and territories as you do in the US, but we still don’t have a national coordination approach to management.

LGould: And is the idea for training to train the medical students at an early level or is it to train people in practice already?

T Swanson: From what I can gather from reading their website and their proposal, it would just be for those who are already registered as general practitioners, and their practice nurses, to a level of wound care standard. They already have their regulatory requirements to be registered, but this would be in addition, basically, so that we know

that the consumables and the assessment are all done on evidence-based requirements and not just individual preferences, and that there's accountability that we use the products appropriately, that is targeted therapy to improve those healing outcomes.

LGould: Okay. And then you've also been part of an international panel that developed a consensus document: The Pathways. What's the plan for disseminating that?

T Swanson: Well, every time that we develop a new initiative, we always publish our methodology and what our goal of these wound care pathways are, and then we get them translated. We also do focus groups within different countries to get the individual requirements. And then now that we have the overarching five step pathway and then the wound specific pathways, I'm currently going through the country here in Australia and speaking to clinicians and localizing it, based on our indigenous and local populations and terminology. So in Australia we don't use the term pressure ulcer anymore, we use pressure injury. And the Pathways say pressure ulcer/ injury. So we're just localizing that. We're bringing more skin tones into the document that we're going to be using. So it's based on rigorous methodology and then localizing to individual countries and then publishing on those findings.

LGould: Yeah. That's fantastic. Maybe you can tell me a little bit about the indigenous population and how to reach that in Australia. It's such a big country with more rural areas than cities.

T Swanson: There is. And there is quite a gap between health care in the rural and remote areas. Certainly for indigenous, we know that there's a significant gap.

And the health statistics for indigenous population is very much like the American indigenous population. We have higher mortality, morbidity, higher rates of diabetes. And so our stats are very similar across the country. What we're trying to do is attract more medical and nursing. The nurse practitioners, similar to the United States, go into these

areas that they can't always attract medical but are supported medically -we have the flying doctor service. But it's still an ongoing strategy, to fill that gap and improve the health statistics for indigenous. But what the government is trying to do is to acknowledge and give voice more to our indigenous population. New Zealand does it fairly well.

L Gould: How did the Australian Medical Association get so involved and interested in wounds?

T Swanson: Well, the general practitioners are the gatekeepers. They see a large proportion of the patients with wounds. And I guess when some of the Medicare funding was removed from the general practitioners, the frustration was voiced that we can't treat the patients the way we want. We can't give them the care that we want. And the Australian Wound

Management Association or Wounds Australia several years ago, funded a study to look at the burden and where those burdens occurred. And they've used that information. They have their strength in numbers as well. And then they put in a proposal. We also

had a change in government that is now more conducive to listening to what the chronic wound is, because it's a hidden area really, isn't it? And it's not as sexy and it doesn't get the publicity. But we have patients that are ten, 15 years with a wound that's not healing. We can do it so much better. But we need that education and funding schemes, which the AMA, fortunately, has been funded, not finalized, but funded up to now.

L Gould: So you say not finalized? I know I've read some of the initiatives, and I thought they were going to start with a diabetic foot. Is that a true statement?

T Swanson: Yeah. So they have the funding, but the implementation hasn't been rolled

out yet for the clinician at the bedside. We still don't understand fully how that's going to be

implemented. But that is one etiology that will have funding. And diabetes in itself is subsidized here in Australia, for their medications with the PBS scheme, married now with initiatives to provide care for DFU. As we know with DFU, it's more than just addressing the wound: It's the off-loading, it's the shoes and the specialized care.

L Gould: Oh that's very good…

**Dr Roch Christian JOHNSON led a pilot project in Benin and Cote d’Ivoire West Africa to develop Community based integrated wound care. In this interview he describes the novel project, the teamwork that was involved in implementing change and the final message that wound hygiene made a major impact when wounds were identified early.**

**This transcript has been edited for clarity while maintaining the dialect of the speaker.**

L Gould: I want to first ask you, how did you get involved in the project to provide wound education to Benin and to Cote d'Ivoire?

RCJ: Yes. As I explained, my professional daily work is management of Buruli ulcer and leprosy. And these two diseases involve a lot of wounds, chronic wounds, and the management of Buruli ulcer includes antibiotics and intervention of disabilities and is performed with a lot of public health intervention especially early detection. The purpose of these activities was to detect early and treat first. And then, we were able to reduce the number of Buruli ulcers. But despite this good result our beds were full of chronic wounds because when we went to the villages, we look at all wounds. And this meant our hospital

was full of wounds. And the facilities in the country, they don't have this possibility to manage properly. So all those who

have wounds came to our centers and so our beds were full of wounds. And they take two months, three months stay at the

hospital and it cost a lot. So our goal, our challenge, was to say, do we wait and patients will come with large wounds or we can

anticipate and see how we can reduce the burden of wounds by management of wounds early in properly prepared centers? It is our challenge. And when we tried to move to this place and see how people manage wounds, we discovered that they have their own processes, their own knowledge.

And it is not possible at all to just go and teach them. We were sure that it would not succeed. This is why we

constituted a multidisciplinary team. In this team we have social anthropology, we have medical doctors, we have nurses,

we have public health officers. And we went to the places and tried to discuss with people first, to know how to understand,

how to learn from them, how they manage wounds and what is the rationale behind this type of management.

And we discovered plenty of things, and it is not possible for us to believe, you know, because for people, for example, in

our minds, management of wounds, we should follow steps and according to the picture of the aspect of the wounds,

we need some type of dressing and things like that. But in the meaning of, in the turf of people, it is not like this at all. For example, they see wounds in their place is like a tomato, so they need to dry the wounds to heal the wounds. So for

them if a wound is dry, this means that it is clean and it will heal. So they have knowledge like that, they have practices like that. For example, for them, if you have wounds and after one month it is not closed, this means that it is due to witchcraft

or sorcerers, a lot of things like that.

So what we tried, so our goal, was to document all these practices and then we classified these practices in what we

call do’s and don'ts. And based on that and taking into account the standard care of wounds, we discussed and came together with a very basic, basic protocol of wound management. And in this protocol, it is mainly based on wound hygiene.

Gould: Yes.

CRJ: Because when, for example, you have wounds, we ask people if you are in your farm and you have wounds, what will you

do? They say we just collect sand and put in the wounds.

LGould: The sand.?

C Roch Johnson: One of the common practices. Or they say, oh, I just went to the bush - I will collect these plants and press the plants in the wounds. And when I arrive to my place, then I will use hot water, very hot water to dress the wounds. So they have a lot of practices like that. They think that in the wounds they have some, what we call in our vocabulary, bacteria. And they need to use the hot water to kill these bacteria. This is why they use very, very, very hot water to clean the wounds. They have a lot of practices like that and it's not possible to change the management without taking this into account. So we learn from them the rationale behind the practices; classify the practices in do’s and don'ts.

L Gould: So some you keep, some you don't right?

CRJ: Yes this is right and then we discuss with them and explain why we propose that this should be don't and why these should be do’s. It was a lot of discussion, a lot of discussion. And then we come to the very basic principles. And in these basic principles we agree on two things: water and soap.

L Gould: And where do you get the water that is clean?

CRJ: Yes. You know in some places thanks to what we call the village eaux des municipalities locales in French, in English, it is water provided by local municipalities. So people have water facilities in their communities. So we can use this water. But after you have the water, another issue is the soap. And this is why we constituted what we call a woman group. And it is constituted by maybe 20 to 25 women. And then we teach them how to make by themselves soap.

So in the place they have both availability of water and availability of soap. In some places, we ask assistance: The Spain Foundation, which is a foundation to provide resources for boreholes and water source with a potable water source.

And we make water available in the places mainly where our diseases are underneath (endemic).

So in the communities where Buruli ulcer and leprosy are underneath, we provide, if not available potable water sources.

L Gould: Wow, that's great.

CRJ: We use this water. Yes. We use these water sources to clean wounds – for wound hygiene. So the main things should be water and soap. And then we need to help them and to teach in a very friendly way.

Because the tool we use, we call flexible PowerPoint with pictures. So we took pictures of different categories of

wounds in the villages. And we follow them step by step. And we look: this is the wound at the beginning. When we use what

we did for our protocol this is the result. Are you convinced with that? Is it treatable for you? What do you want to change? And with this dialog we come up with very basic and practical tools. And then we validate these tools in a cohort study to demonstrate if we can have good results thanks to these materials. After validation…Yes, please.

L Gould: Oh, with the PowerPoint, was that on a computer or did you have posters or how were you be able to portray them. CRJ: We put, you know, in the village, we put in the village, then what we call, (French words) a small generator, with computer, and then we project.

Gould: Okay. Yes.

CRJ: Yes. So we have a kit, a sensitization kit comprising with a small generator, a computer, a screen,

and we make appointments with villagers. It is mainly during nights when they come from the farm, because during the

day they farm and they are not available. So the appointment was when they come to the village from the farm.

Then we went to the place to discuss with them and try to make our community awareness about wound management.

So this is how we proceed to perform this wound education. And we are happy to see that more than 90% of the wounds, when we start very early, the wounds were able to heal. So when we demonstrate that it is possible, so people adapt to what we propose. And it was a sort of what we call, it is our common activity that we in our mind, we don't travel to

the places to teach people. In our mind, we went to the villages to discuss with them and come together to a

solution of the problem ___. It is our strategy.

L Gould: Good. Now, I'm sure you met some big obstacles, right? Some people that wouldn't change some things. I think there was some antibiotic use. What were the biggest things that were problems?

CRJ: Yes. The problem, the biggest thing is culture. Because they believe that the wound is due to witchcraft, sorcery. If the wound is a chronic wound, this means that it is not a natural wound. So this was a big issue. And also you know, snakebites is also an issue. And the wounds due to snake bite was also a main problem in these places. And this was one of the challenges we faced. What people think about wounds was one of our challenges.

So to change this, is to explain that a wound is manageable. It was an issue, it was a challenge. It was. Yes.

L Gould: Now you said that you mostly worked with a group of women. What about the men? Probably the men get a lot of the ulcers. How were they receptive to your teaching?

CRJ: Yes. The group of women is mainly for soap.

Okay. Yes. But in the place we worked we constituted what we call informal group discussion. In this group, this routine, it is

comprised with men, women, patients, relatives of patients, and we teach them our protocols and the rule is to support the activities in the place. So when people have a wound, they can advise them because they know how to proceed.

L. Gould: So you're teaching multiple levels.

CRJ: Yes. Multiple levels because we have the community level. But after the community level it is very, very, very important to also train nurses. Because the nurses, as well as the communities, they should be on the same line. Let me say something. Okay. Say we have taught people that management of wounds is not just to put antibiotics on the wounds.

You know we teach people in the community that is in the don’ts group. And we also teach what we

call alarm sign: when you start to clean your wounds, what will be the danger sign? And if you

have this sign, you should directly go to the hospital. For example, if the wound is painful, if the wound is bleeding, for example you need to go to the hospital. But when we teach them that the main activity, the main issue of wound management is not antibiotics but wound cleaning. And then when they go to the centers and the nurses put antibiotics on the on the wounds, this is in opposition to what we explain to them in the community. This is why it is also very important to train nurses so that the communities as well as nurses will be on the same line, yeah, with the same philosophy. So we try to put this into place. And also one of the most challenging is also to change the mind of nurses because they have their way to dress wounds.

And to change their mind was not easy at all. So it was a lot of dialogue, a lot of discussion, a lot of training. Not training from professor to the student. No, we need to sit, to sit together, discuss, demonstrate. Sometimes you need to come many times and then they realize that they can have a good result. And we set up this strategy. So at the end we were really happy.

L Gould: You got very good results. So how is your project funded?

CRJ: Yes. You know, for this project in Benin and Cote d’Ivoire we received funds from two foundations. The first foundation is a UBS foundation. UBS is a bank in London and they have a foundation. So we received 50% of our funding from this foundation and the second 50% was provided by the Spain Foundation called Anesa. So our project was funded by both UBS Foundation and Anesa foundation.

L Gould: So then what do you do next to keep it going or to expand?

CRJ: So you know, but what we try to do is that, sustainability is a big issue in our place. Sustainability. So I have started my professional activities in 1988, in Benin. And I can tell you that sustainability in our place remains to humans, human resources. So if you want to sustain your program, you need to treat people. So that at the end of the project they should continue. The second issue of sustainability is institutions. It is very important when you want to run a project, to link these projects to a stable institution and make them active so that they will own the project. And they will continue. If not it will not work. This is why before we start the project, when we conceived the project, we discussed with the National Cultural Program the Buruli ulcer and leprosy, the National Cultural Program both in Benin and Cote d’Ivoire. So this program was the owner of

the project and we are the facilitator. Yeah. So we are facilitators but the owner of the project was the National Cultural Program. And we made our project very cost efficient because, as I explained to you, we didn't provide imported dressings but basic dressings like gauze, water, soap. And what we call Verticalite - you know I don’t know how to explain in English, but it is a local, local, verticalite... How I can say - I will send you the name later. Right. It is in the market.

Shea butter. It is shea butter. So it is available on the local market. So we use all these basic things for wound dressing. And at the end, it is also possible to the communities and National Cultural Program to continue what we put into place. And, actually, I'm very happy to tell you that actually there is a request from Togo, another country. They asked us to come and teach them and share our material. And to do the same for wound management in their place. So in Benin, as well as in Cote D’Ivoire this strategy is expanded to more than five districts in Cote d’Ivoire more than five districts in Benin. So it still continues.

L Gould:That's great. You know, we face many of the same problems in the US in terms of wound hygiene, using antibiotics, trying to teach a lot of people, especially in rural areas. And I think the message to keep it simple and to catch them early is so important. We hope your work continues. And, we like we would like to stay in touch and, learn from you how to bring good education to the people and really using what the people know.

CRJ: Right. And what is available, what is available in that place.

**Simone McConnie provides a passionate discussion of the importance of podiatry combined with wound care education and how the Step by Step program was translated from success in Tanzania to the Caribbean Islands. She also explains the need for financial and administrative/government support to develop and sustain a program to prevent amputations in patients with diabetic foot ulcers.**

**(Transcript edited for clarity)**

L Gould: Simone, let's start off with you telling us a little bit about yourself and how you got into this field.

SM: From the beginning? I would say that my whole field of wound care and podiatry really started because I just wanted to be a podiatrist, because my country needed podiatry or ‘chiropody’, to help improve and save limbs. So, you know, I’m coming from an island where we have a lot of general practitioners, and I thought in my head I didn't want to be a general practitioner. I wanted to do something that was a little bit different. I didn’t want to be a physio-, I didn't want to be an occupational therapist. And I heard a speech from my Minister of Health who said that, you know, we needed chiropody or podiatry. And I did some research and realized, yeah, this is new and they say they need us. So I thought, that's rocket science. Yeah, got that! So I was young enough that if I got into it, I felt that if I didn't like it I could change.

But this is 31 years later and I'm still doing it, so I love it. It's a very rewarding profession. And then my journey, you know, really started because of the love of the diabetic foot and how easy it is to really do prevention and salvage. But yeah, we find it very challenging in our Caribbean region because the small things are not put in place.

So I worked with Ali Foster and Mike Edmonds at King's College. I did a little stint with them, and at that time they were shining as one of the first clinics in the UK that were doing a multidisciplinary team where you had podiatrists and you had internists, that were literally, they were saving limbs.

And I came by all bright eyed and bushy tailed to Barbados, thinking that was going to be quite easy, because Barbados wanted podiatrist. And I had this little bit of zest. And if I came back and I found a doctor, surgeon or, you know, someone that I can work along with we could repeat the same thing and we could just reduce amputations.

But I was rudely awakened to the fact that life is not that easy. And of course, there's politics, and there's a lot about other things that are involved. And there was a lot of rejection, a lot of abuse, a lot of aggression, a lot of negativity towards having

podiatry, recognition of what a podiatrist is, understanding the need for podiatrists. And it's almost like - it was quite weird because, you know, it's kind of like on one hand you're saying this is what we need and then on the other hand you get it, and then you go, oh, I don't know who you are. So it was almost like calling for something that you needed that you didn't even know that you needed or understood what that need was.

Anyway, that situation really hasn't changed very much in the last 31 years, I would say. Because I had a recent discussion with one of our chief medical officers, you know, he was saying to me that, you know, we are trying to help, but they're not asking for help. So as a result, you know, to me, we're still where we are, but yet in the speech almost

35 years ago, they were asking for help.

So it's one of these political spirals, I guess. But moving on from there, the reality is people are suffering. And, you know, my skin has gotten thicker over the years and I just push on really on the behalf of patients, because that's really what breaks my heart more than anything, which is seeing the suffering. So evolving from there, I guess

Alethea Foster became involved in a program that was developed in Tanzania by Doctor Abbas. And that was birthed out of him realizing the importance of a multi-disciplinary team. And it was named the Step by Step. And, you know, it was really about

improving the lives of people and reducing amputations in the developing world. And the Caribbean as part of the developing world.

So I would say that started in Dar es Salaam. And people like Karel Bakker and Alethea Foster and Kristein von Acker, and there were a whole lot of very, knowledgeable people that were able to basically break their barriers with regards to getting rid of the egos, getting rid of anything that surrounds that and work together as a team.

And they were able to achieve 50% reduction in amputations just by implementing a team approach. And that then was multiplied. So it worked there and then Doctor Abas was able to develop other clinics around Africa that were doing the screening programs and helping to identify the at risk foot at an earlier stage to help reduce amputations.

L Gould:So this is education for the non-wound specialist?

CM: Yes, education for the non-wound specialists.

And I think it was also recognition that the diabetic foot dovetails with wound care. So if you're doing diabetic foot and you don't have a good knowledge of wound care then you're probably, not treating, you're not doing it well. You probably

probably can identify the at-risk foot. But when it comes to the other end of salvaging a problem foot, you will have challenges if you're not doing wound care.

So then my love for wound care and diabetic foot developed. I did some training and then more training in wound care. And I actually recognized that podiatry is probably one of the only professions that we are taught wound care as part of our training. You know, I've learned over the years talking to other general practitioners that they don't do anything to do with wound care when they're training.

And I was surprised, because obviously, when you go to a general practitioner as a primary care provider and you have a problem, they're expected to heal your venous ulcer, they're expected to heal your bed sore, they're expected to heal your diabetic foot ulcer. And they don't have the tools. They don't have the knowledge. They don't have the education.

So, you know, I would say that over the years, I've had different challenges with regards to different programs. So the Step by Step program came on board and we did a basic and advanced, which includes wound care.

So along with identifying the at-risk foot, which is your neuropathic foot, or arterial disease, you're also looking at how can we heal this limb that does presents with an ulcer through debridement and offloading, etc? And that opened another jar of worms. Because you then realize that you don't actually have those resources. You know,

across the Caribbean, we maybe have about 20 podiatrists. And when you're looking at the across the region itself, 20 podiatrists is nothing. It's a drop in the ocean. So as a result, as I have moved from one island to the other, I have noted that every island I've been to: Antigua, Saint Kitts, Dominica, Grenada: they all consider themselves as the amputation capitals of the world because of how many amputations they see. And for me, because I'm a lover of wounds and a lover of wound care, you know, give me that most difficult wound to heal and I got you. Because that is a challenge, right? You’ve got a wound like that, it is a challenge. It's almost like a mystery because initially, when you get a wound like that, you've got to play detective, because you have to figure out, okay, what's going on with this patient? What's their underlying conditions, what's their living conditions like, what are they eating? What's the skin type like? You know, you're asking yourself a thousand questions at that one glance. You know, what's the wound like? How long has it been here? You know, so you're going through there, you're processing the whole thing, and you're trying to tick all the boxes to get it right.

And I would say that that is missing, that is missing from our healthcare system. Because I feel that if you are treating a wound and the wound is not healing, you should have some kind of framework where you're thinking, okay, it's

been six weeks, it's been a year, I’ve been dressing this foot. Something is wrong.

It's not just taking a dressing off. If you're taking a dressing off, putting another dressing on, if you're diluting povidone iodine, if you're doing any of these things… If you're picking up a dressing because a drug rep came in and said, oh, this is new and it works really well, or if you're injecting growth factors into a wound that you've had for a long time, but you just figured, you know what let me just try some growth factors on this, because I've been putting betadine and gauze on it for a long time. So I'm going to jump straight to growth factors. If you're doing that, you're not doing wound care. You're not even started to do wound care. All you're doing is - you're actually searching for help, because you're not actually addressing what that patient requires in order to bring that wound to closure. And yes, wound healing takes time. It takes love. And I think the thing that goes with wound care is that it's not just about the care of the wound either. It's the creativity behind it. It's almost like an art. I think that wound care is an art.

And as you do more and more wound care, there are certain things you identify pretty early on. Yes: The odor coming from the wound, the appearance of the wound. There are just certain things you can look at that wound and, you know, okay, that is definitely probing to bone. That has to have a bone somewhere in there. You know that slough is not the right color. You just know when that wound is wrong.

So when it comes to the Step by Step, and we went back to that, I would say, yes, it didn't cover that in detail, but it did teach you about debriding the wound and the impact of different types of dressings: whether you're going to use an alginate or foam or a combination, you know, so you had an appreciation of when you're changing a dressing, what kind of dressing you wanted to be thinking of.

L Gould: I'm going to interrupt you. Let's do this: We’re going to go back to the Step by Step program so that you can, you know, kind of outline how it went from Tanzania to the islands. CM: Okay.

L Gould: And, kind of short. And then I'll ask you again, what are the obstacles and is there a way to make it sustainable? I love listening to you. We could go on forever. Let's try this. Okay. So, Simone, you've been very instrumental in bringing the step by step program to the Islands. Can you tell me how it went from Tanzania to Barbados or other islands and what the impact has been?

SM: Okay, so first of all, small correction. I wasn't the only one was really part of bringing it to - it was a combination of a team, I guess. I think I was specially earmarked by doctor Karel Bakker and Ali Foster to be someone to carry the torch, after they kind of initially created it, and bring it here. And I would say the program and Tanzania's success with Karel Bakker and Ali Foster, showed that they could reduce amputations by 50% with implementing the screening programs and the prevention programs.

So that was then the foundation of the educational model that was created for the training for the Step by Step. And a model was created, you know, with people like Stephen Morbach and Doctor Kristien Van Acker. So even that process was a team approach. It was not anything that was me instrumental doing it.

I guess I've been just instrumental in trying to keep the fire going. And I would say that the Rotary clubs of the Islands then came on board and because the World Diabetes Federation, I think, they didn't realize how long and how much effort it was really going to take to be able to translate that in the Caribbean islands.

So they then kind of started exiting, we needed a continuum of funding. So the International Diabetes Federation kind of came on board, and then the Rotary clubs across the islands came on board, and they did like matching grants in order to help sustain the program in the islands. I would say the successes of the program was to build awareness.

So initially I would say if I had to reflect, it was building awareness that something can be done to reduce amputations. Then it was the importance of government buy-in, on the level of standard of care. So I would say the only island that bought in on the standard of care was Dominica. Again, it is still a political game because I feel that Dominica’s success was because at the time, their Minister of Health, said if he didn't facilitate the ability to reduce the amputations, you know, by a certain timeline, he was willing to reduce his salary by 50%.

So it was successful from the perspective that obviously all of the chess players were moved in order to facilitate it. And they actually did an amazing job because I think after one year they reduced their amputations by 23%. And I think after

about three years they reduced amputations by 72%. So it just goes to show you that it does work if it's implemented. But then the challenge that we have moving forward is the sustainability. So even now it actually has just been rebirthed, not necessarily as a step by step, but it has been rebirthed as a training program, with foundations in

wound care and identification of the at-risk foot. So that's happened in Saint Kitts and Nevis and in Antigua. So I've just come off training with them, doing just a revamp of it in a different model. The different model now is to bring

some kind of sustainability and some kind of resources that would help with the continuum. And those resources I think need to be podiatrists. There's no two ways around it. Yes, we can train nurses. Nurses are supportive. But unless you are a podiatry assistant with wound care, or you are a podiatrist - If you're dealing with a foot, you really need to have a podiatrist on your team. If you don't have a podiatrist, you're still going to

fail. And you have to have the wound care specialty, and you have to work as a team. I think I think the challenges that we have right now is recognition of the fact that we cannot do all these things on our own and we need a team in order for it to be done effectively. While your management of the diabetes needs to be done by your internal medicine specialists or your GP. Whether the management is helped with the nursing, you may have your prosthetist or orthotist dealing with special footwear, especially if the patient has foot gait challenges. You need the podiatrist to be looking at the things that we're going to see that no one else is going to see.

And unless you've trained to be a podiatrist, although we can train for the step by step, we can train you to identify certain things, we cannot train you in a in a four-day program how to be a podiatrist.

L Gould: That's great. That's great. I can use this. So I think, one of the plans for this panel is then to develop a group of invested people, you know, take the learnings from the

panel, take the feedback, and try to develop a program. Some of the feedback we're getting is people like a hybrid program, you know, now that online is become much

more acceptable, a combination of online and then skills. And, you know, try to develop that with the support of the Wound Healing Society Past Presidents, and whatever we glean from our session in May, and then, next year at EWMA, present updates.

SM: Oh, that sounds good. Yeah. So, so I would say one of my other areas of trying to find a way of change has been to bring on board people who are similar thinking. So bring on board persons who are interested in the Caribbean.

Those who are interested in helping me to develop something that is sustainable, not necessarily the I am holding the reins, but that you're kind of coming alongside and throwing in your $0.03 and saying, well, this is what we can do. This is what we might want to do. This is how we can help you, and this is what we think you should do. And there Is some support right now from Wounds Canada. There is some support from the Canadian Podiatric Medical Association.
